# Identifying the driver miRNAs with somatic copy number alterations driving dysregulated ceRNA networks in cancers

**DOI:** 10.1186/s13062-023-00438-x

**Published:** 2023-11-22

**Authors:** Renjie Dou, Shaobo Kang, Huan Yang, Wanmei Zhang, Yijing Zhang, Yuanyuan Liu, Yanyan Ping, Bo Pang

**Affiliations:** College of Bioinformatics Science and Technology, Harbin Medical University, Harbin, 150081 Heilongjiang China

**Keywords:** Somatic copy number alteration, Driver miRNAs, ceRNA network, Dysregulation, Prognosis

## Abstract

**Background:**

MicroRNAs (miRNAs) play critical roles in cancer initiation and progression, which were critical components to maintain the dynamic balance of competing endogenous RNA (ceRNA) networks. Somatic copy number alterations (SCNAs) in the cancer genome could disturb the transcriptome level of miRNA to deregulate this balance. However, the driving effects of SCNAs of miRNAs were insufficiently understood.

**Methods:**

In this study, we proposed a method to dissect the functional roles of miRNAs under different copy number states and identify driver miRNAs by integrating miRNA SCNAs profile, miRNA-target relationships and expression profiles of miRNA, mRNA and lncRNA.

**Results:**

Applying our method to 813 TCGA breast cancer (BRCA) samples, we identified 29 driver miRNAs whose SCNAs significantly and concordantly regulated their own expression levels and further inversely dysregulated expression levels of their targets or disturbed the miRNA-target networks they directly involved. Based on miRNA-target networks, we further constructed dynamic ceRNA networks driven by driver SCNAs of miRNAs and identified three different patterns of SCNA interference in the miRNA-mediated dynamic ceRNA networks. Survival analysis of driver miRNAs showed that high-level amplifications of four driver miRNAs (including has-miR-30d-3p, has-mir-30b-5p, has-miR-30d-5p and has-miR-151a-3p) in 8q24 characterized a new BRCA subtype with poor prognosis and contributed to the dysfunction of cancer-associated hallmarks in a complementary way. The SCNAs of driver miRNAs across different cancer types contributed to the cancer development by dysregulating different components of the same cancer hallmarks, suggesting the cancer specificity of driver miRNA.

**Conclusions:**

These results demonstrate the efficacy of our method in identifying driver miRNAs and elucidating their functional roles driven by endogenous SCNAs, which is useful for interpreting cancer genomes and pathogenic mechanisms.

**Supplementary Information:**

The online version contains supplementary material available at 10.1186/s13062-023-00438-x.

## Introduction

Somatic copy number alterations (SCNAs) are common genetic alterations affecting cancer development, which enable change in the genetic regions to affect cancer-related transcriptome activation or inactivation [1], while its regulatory capacity is not only on protein-coding regions but also on noncoding regions. SCNAs also influenced expression levels of genes outside the amplified/deleted regions [2]. The network modeling of global gene expression identified a glioblastoma-associated CNA-driven network, and indicated that driver CNAs could transfer variation information across topological networks, affecting adjacent or downstream interacting genes [3]. In addition, driver CNAs could cause abnormal activities of biological pathways by interfering gene expression in the pathways [4], or spread mutation information along the topological network of the pathway and cause abnormal pathway function of cancer individuals [5]. However, these studies are still limited in exploring the direct effects of SCNAs on tumor progression.

MiRNAs are non-coding RNAs that regulate mRNAs by targeting the 3 'UTR of genes and underlie post-transcriptional regulation of protein-coding genes (PGs). Change in miRNA expression is likely to affect the degree of target regulation and cellular homeostasis [6]. The hypothesis of competitive endogenous RNA (ceRNA) uncovered a more complex pattern of cross-regulations among miRNAs, lncRNAs and mRNAs—the ceRNA network [7]. The dynamic balance of between miRNAs and their target regulatory relationships is important for maintaining the normal biological functions of cells. Abnormal expression of miRNAs could disrupt the balanced ceRNA networks. The lncRNA-CDC6 could function as ceRNA via directly sponging of miR-215, and overexpression of the lncRNA-CDC6 further regulated CDC6 to promote the proliferation and metastasis of breast cancer cells [8]. LncRNA FAL1 acted as a sponge of mir-637, whose high expression promotes the carcinogenesis of colorectal cancer cells by downregulating the expression of tumor suppressor gene NUPRL [9]. Xu et al. reported that copy number variations (CNV) were also important factors in the dysregulation of ceRNA interactions [10]. Subsequently, many studies began to explore the driving effects of CNVs on the ceRNA networks. Ding et al. constructed a ceRNA network of CNV-driven lncRNAs in lung squamous cell carcinoma patients [11]. Zhu et al. performed a comprehensive analysis of the dysregulated ceRNA network caused by copy number variation-driven lncRNAs in breast cancer [12]. Loss of mir-4484 acted as a tumor suppressor and promotes glioblastoma development and progression [13]. However, the effect of endogenous expression perturbations of miRNA caused by SCNAs on ceRNA networks was less explored.

**Fig. 1:**
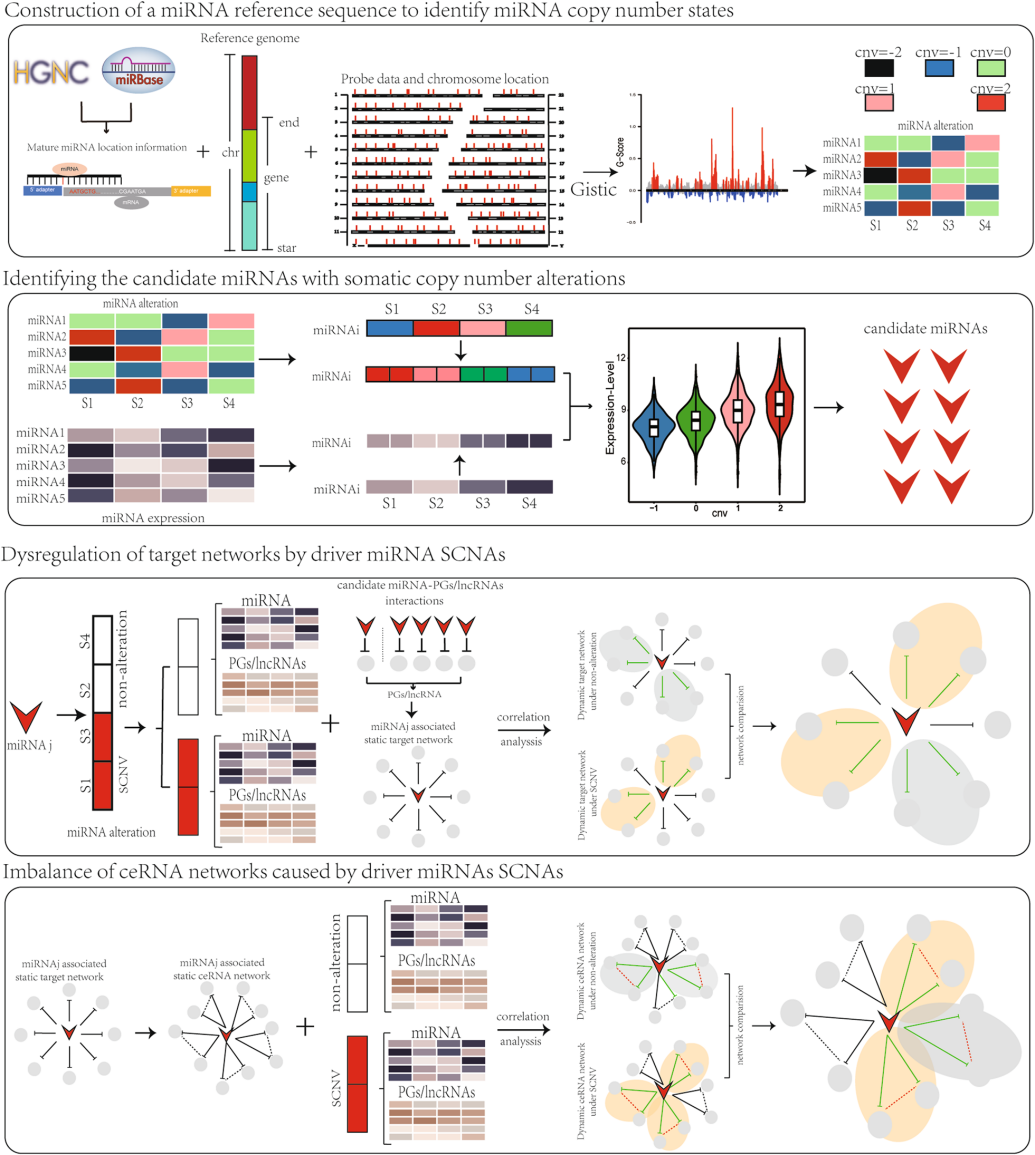
The workflow of the integrative strategy for identifying SCNA-induced dysregulated ceRNA networks

In this study, we provided a method to identify the driver miRNAs and characterize the functional roles of miRNA SCNAs based on the dysregulation of ceRNA networks (Fig. 1). Firstly, we constructed the SCNA profiles of miRNA based on miRNA genome location data and segment data. Secondly, we integrated the SCNA profiles and expression profiles of miRNA to identify candidate driver miRNAs. Third, we identified driver miRNAs whose SCNAs drove their own differential expression and further caused the imbalance of target networks or dysregulation of target gene expression. Then, based on the miRNA-target network, we constructed dynamically activated ceRNA networks in each SCNA state and identified the dysregulated ceRNA networks driven by the SCNAs of driver miRNAs.

We applied our method to breast cancer (BRCA) identified 29 driver miRNAs and identified three distinct influence patterns of SCNAs of driver miRNAs on their mediated ceRNA networks. High-level amplifications of four driver miRNAs in 8q24 characterized a BRCA subtype with poor prognosis and contributed to the dysfunction of cancer-associated hallmarks in a complementary way. We also applied the method to other three cancer types (OV, LUAD and LUSC) and compared the difference of dysregulated ceRNA networks driven by SCNAs of driver miRNAs.

## Materials and methods

### Materials

#### The multi-omics datasets of breast cancer

We collected copy number data, expression profiles of miRNAs, protein genes and lncRNAs, and clinical data of breast cancer (BRCA) samples from The Cancer Genome Atlas (TCGA, https://www.cancer.gov/about-nci/organization/ccg/research/structural-genomics/tcga).

#### Copy number data

The copy number segmental data of 1102 TCGA BRCA samples were downloaded from TCGA by using the R package TCGAbiolinks.

#### The expression profiles of miRNAs and PGs

The expression profiles of miRNAs and protein genes (PGs) of BRCA samples were downloaded using TCGAbiolinks. The expression level of each mature miRNA from a specific chromosomal location was calculated as total read numbers from the corresponding chromosomal location, and then transformed into RPM (Reads Per Kilobase of exon model per Million mapped reads). The log2 (RPM + 1) was used for subsequent analysis. The expression of PGs for the BRCA were detected using RNA sequencing (RNA-seq) technology. The expression levels of PGs were calculated by FPKM (Fragments Per Kilobase of exon model per Million mapped fragments). Biotools.fr was used to convert gene names from Ensemble ID to symbol and calculated the average values of same gene as the gene expression level. The log2 (FPKM + 1) was used for subsequent analysis.

#### The expression profile of lncRNAs

LncRNA expression profiles of 837 BRCA tumor samples were obtained from the Atlas of Noncoding RNAs in Cancer (TANRIC) (http://ibl.mdanderson.org/tanric/_design/basic/index.html) [14]. The expression level of lncRNA was measured by RPKM. We used BioTools.fr to convert gene names from Ensemble ID to symbol. The log2 (RPKM + 1) were used for further analysis.

#### Clinical data

The clinical information of 1097 BRCA samples including survival time was downloaded from TCGA.

813 common BRCA samples, which were detected in all of these four types, were used for identifying driver miRNAs with copy number alterations.

#### miRNA target data

We downloaded the miRNA–mRNA target interactions from starBase v2.0 [15] (https://starbase.sysu.edu.cn/index.php) and the miRNA–lncRNA target interactions from lnCeDB [16] (http://gyanxet-beta.com/lncedb/). We used miRNA–mRNA/miRNA–lncRNA target interactions to construct the static ceRNA networks for each miRNA.

#### Hallmark gene sets

The gene sets of 50 cancer-related hallmarks were downloaded from MSigDB [17] (http://software.broadinstitute.org/gsea/msigdb/index.jsp) which contains 3 cell components hallmarks, 6 growth and development hallmarks, 3 DNA damage hallmarks, 7 immune system hallmarks, 7 metabolism related hallmarks, 5 Pathway related hallmarks, 6 cell proliferation hallmarks, and 13 signal transduction hallmarks.

### Methods

#### Constructing the copy number profile of miRNAs

In order to obtain the copy number profiles of all mature miRNAs, we first prepared miRNA reference information in strict accordance with the reference genome (Hg. 38. CSC) information used in GISTIC2.0 [18]. For the same mature miRNA expressed from the different chromosome locations, we extracted the start and end positions of mature miRNA by integrating the location information of expressed miRNAs in 1202 BCRA samples and added the location information of the mature miRNA into the reference genome through MATLAB. Based on the reference information of mature miRNAs, we used GISTIC 2.0 to detect copy number profiles of miRNAs which included continuous and discrete copy number matrixes. In the discrete copy number matrix, five values are used to measure the copy number states: homozygous deletion (− 2), heterozygous deletion (− 1), diploid (0), gain (1) and high-level amplification (2).

#### Identifying the candidate miRNAs

Candidate miRNAs were defined as whose SCNAs could significantly and concordantly affect their own expression. For each miRNA, we grouped BRCA samples into five subgroups based on discrete copy number status (− 2, − 1, 0, 1, 2) of the miRNA and filtered out subgroups with less than 5 samples. To detect which miRNA’s copy number states could significantly and concordantly affect their own expression, we used Student's t-test to pairwise compare the miRNA expression between different subgroups in pairwise (− 1 vs − 2, 0 vs − 1, 1 vs 0, 2 vs 1, *p* < 0.05). We expect that the expression level of candidate miRNA should be significantly higher in high copy number subgroups than that in low copy number subgroups.

#### Identifying the driver miRNAs

We analyzed the driver effect of SCNAs of candidate miRNAs from two aspects: dysregulation of miRNA-target networks and differential expression of miRNA targets.

##### Dysregulation of activated miRNA-target networks

For each candidate miRNA, we constructed a static miRNA-target network according to the interaction relationship between the miRNA and its targets from starBase and lnCeDB.

For each copy number state, we integrated expression profiles (including PGs, miRNAs and lncRNAs) and the candidate miRNA mediated static miRNA-target networks to construct the activated candidate miRNA-target network. We calculated the Pearson correlation coefficients (PCCs) between the candidate miRNA and their targets. The candidate miRNA-target pairs were considered active only if their PCCs were significantly negative at FDR = 0.05. All active candidate miRNA-target pairs formed the activated candidate miRNA-target network.

In order to investigate the effects of the SCNAs of candidate miRNAs on the miRNA-target networks, we compared the activated candidate miRNA-target networks from different SCNA states (− 2, − 1, 1, 2) with that from copy number status of diploid. The differential miRNA-target pairs formed the dysregulated miRNA-target networks driven by each copy number state.

##### Differential expression analysis of miRNA targets

To analyze whether SCNAs of candidate miRNAs could drive the abnormal expression of its targets, we used Student's t-test to compare expression levels of targets among different copy number states (− 1 vs 0, 1 vs 0, 2 vs 0 and 2 vs 1). Due to inhibiting effects of miRNA on the gene expression of targets, the differential expression targets were identified as significantly up-regulated targets for comparison of − 1 versus 0 at fold change (FC) > 1.2 and *p* value < 0.05, and the differential expression targets were identified as significantly down-regulated targets for comparisons (1 vs 0, 2 vs 0 and 2 vs 1) at FC < − 1.2 and *p* value < 0.05.

#### Constructing the dysregulated ceRNA networks of driver miRNAs

##### Constructing the static ceRNA networks for driver miRNAs

For each driver miRNA, we extracted the targets (PGs/lncRNAs) of the driver miRNA from the miRNA-target networks. The driver miRNA and each pair of targets formed a ceRNA triple unit. All ceRNA triple units were assembled into a static miRNA-mediated ceRNA network.

##### Constructing the activated ceRNA networks under each copy number state of driver miRNAs

For each copy number state of the driver miRNA, we integrated expression profiles of miRNA, protein genes and lncRNAs and the driver miRNA-associated static ceRNA network to construct the activated ceRNA network. For each ceRNA triple in the static ceRNA network, we calculated the PCCs among miRNA and ceRNAs. The ceRNA triple was considered as active only if the PCC between driver miRNA and targets were significantly negative and that between targets was significantly positive at FDR = 0.05. All activated ceRNA triples formed the active driver miRNA-mediated ceRNA networks under the specific copy number state.

##### Constructing the dysregulated ceRNA network driven by the SCNAs of driver miRNAs

To explore the driver effect of the SCNAs on driver miRNA-mediated ceRNA network, we compared the activated ceRNA networks from different SCNA states (− 2, − 1, 1, 2) with those from diploid state. We defined the dysregulated triples as the inactivated or newly activated triples in SCNA states. Then, all dysregulated ceRNA triples constituted the dysregulated ceRNA networks affected by the SCNAs of driver miRNAs.

#### Survival analysis

To test whether SCNAs of driver miRNAs were significantly associated with BRCA prognosis, we compared survival times of subgroups under different copy number states of driver miRNAs using the Kaplan–Meier method and log-rank test.

To test the association of high-level amplification of the specific genome region with BRCA prognosis, cancer patients were subdivided into three subgroups: subgroup I including patients with at least one high-level amplification of driver miRNAs in this region, subgroup II including patients without high-level amplifications but with heterozygous deletion or gain of in driver miRNAs, and subgroup III including patients without any SCNAs of driver miRNAs. The Kaplan–Meier analysis and log-rank test were used to estimate the significance of survival differences among subgroups.

## Results

### Identifying the candidate miRNAs with somatic copy number alterations

**Fig. 2:**
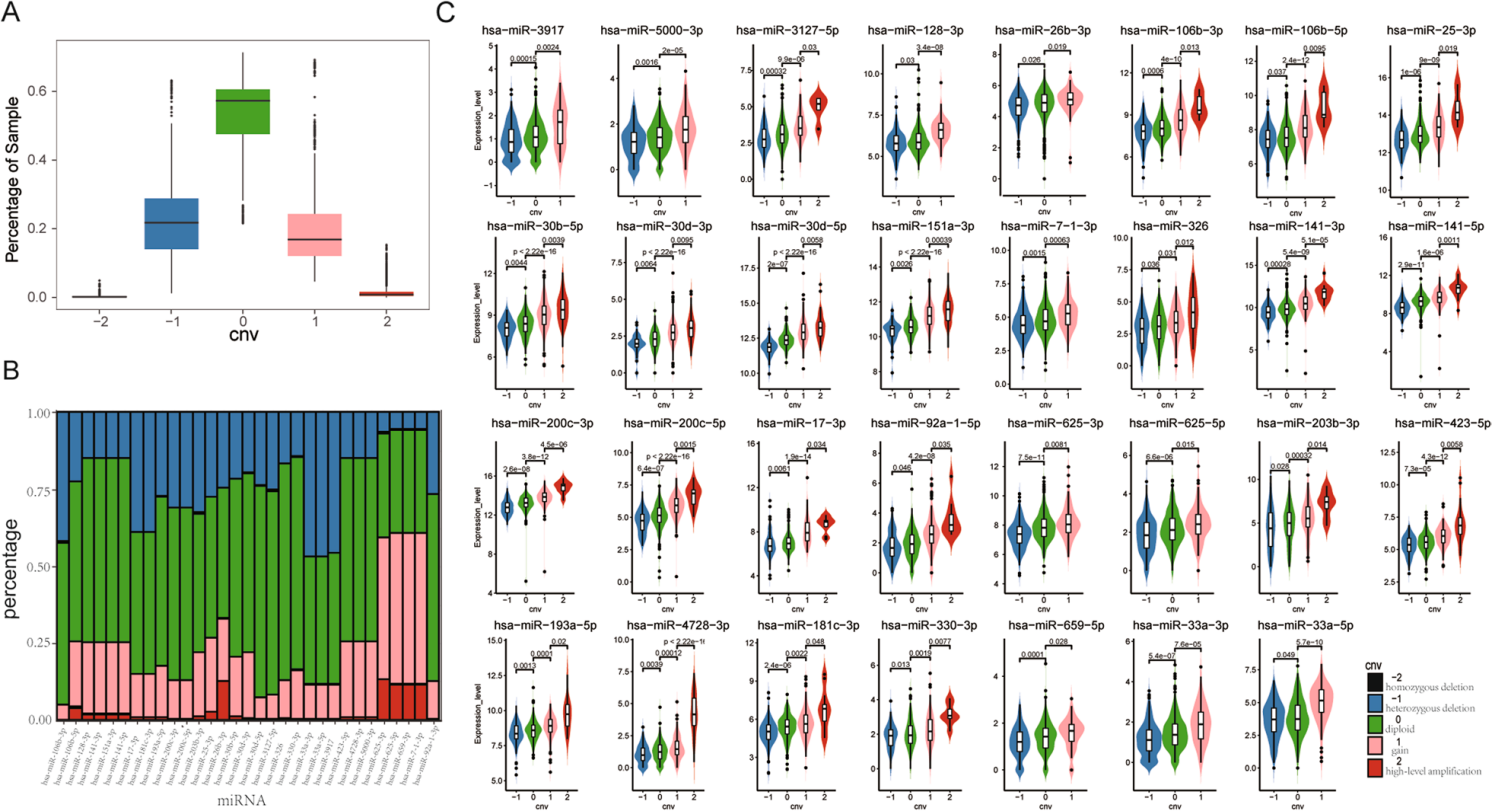
Somatic copy number alterations of candidate miRNA. **A** Sample percentage of candidate miRNA copy number states. **B** Frequency distribution of the five copy number statuses of candidate miRNAs in BRCA. **C** Expression levels of candidate miRNAs varied significantly and consistently with copy number changes

The start and end genome positions of 2405 mature miRNAs were obtained by processing the position information of mature miRNAs whose expression levels were detected in 1102 TCGA BRCA samples. By combining the genome positions of mature miRNAs in the format of the reference genome (H38), we applied GISTIC 2.0 to analyze the copy number segment data from 1202 TCGA samples and constructed the somatic copy number profile for 2405 mature miRNA. This profile contained five copy number states [including homozygous deletion (− 2), heterozygous deletion (− 1), diploid (0), gain (1) and high-level amplification (2)]. We calculated the frequency distribution of the five copy number states of miRNAs across the BRCA population. We found that the diploid state of miRNAs dominated, whose frequency ranged from 48 to 61% (Fig. 2A). The heterozygous deletion frequency and gain frequency of miRNAs were comparable (frequency ranged from 15 to 25%). However, the high-level amplification frequency of miRNAs was generally low, with only a few of miRNAs showed relatively high frequency (such as has-miR-4728-3p with 12.7% and has-miR-151a-3p with 13.3%). Moreover, the homozygous deletion frequency of miRNAs was extremely low, and even a considerable proportion of miRNAs had no sample with the homozygous deletion state (Fig. 2A). For example, the frequencies of different copy number states of has-miR-141a-5p were 1.97% for high-level amplification, 23.25% for gain, 60.02% for diploid, and 14.76% for heterozygous deletion, respectively.

We combined the somatic copy number profile and expression profile of miRNAs in 813 common BRCA samples to identify candidate miRNAs whose somatic copy number alterations could affect their own expression levels. By comparing the expression levels of miRNAs under different copy number states with Student's t-test at *p* = 0.05, we identified 31 candidate miRNAs whose expression levels significantly increased with the copy number state changed from heterozygous deletion to high-level amplification (Fig. 2B, C). For instance, the expression levels of has-miR-141a-5p significantly and consistently elevated with increasement of copy number of has-miR-141a-5p (*p* = 2.9e−11 for diploid vs heterozygous deletion, *p* = 1.6e−06 for gain vs diploid, and *p* = 0.0011 for high-level amplification vs gain). These results suggested significant copy number dosage effect of miRNAs which may further contribute to BRCA carcinogenesis by disturbing their targets and subsequent cascade effect.

### Dysregulation of target networks by SCNAs of driver miRNA

We performed two analyses to explore the driver effect of miRNA SCNAs in BRCA. The first analysis aimed to determine whether miRNA SCNAs could significantly cause reverse change in the expression level of miRNA targets. The second analysis aimed to test whether miRNA SCNAs could disturb the miRNA-target relation networks. The miRNAs were identified as driver if their SCNAs could either disturb the miRNA-target relation networks or significantly affect the expression level of their targets.

**Fig. 3:**
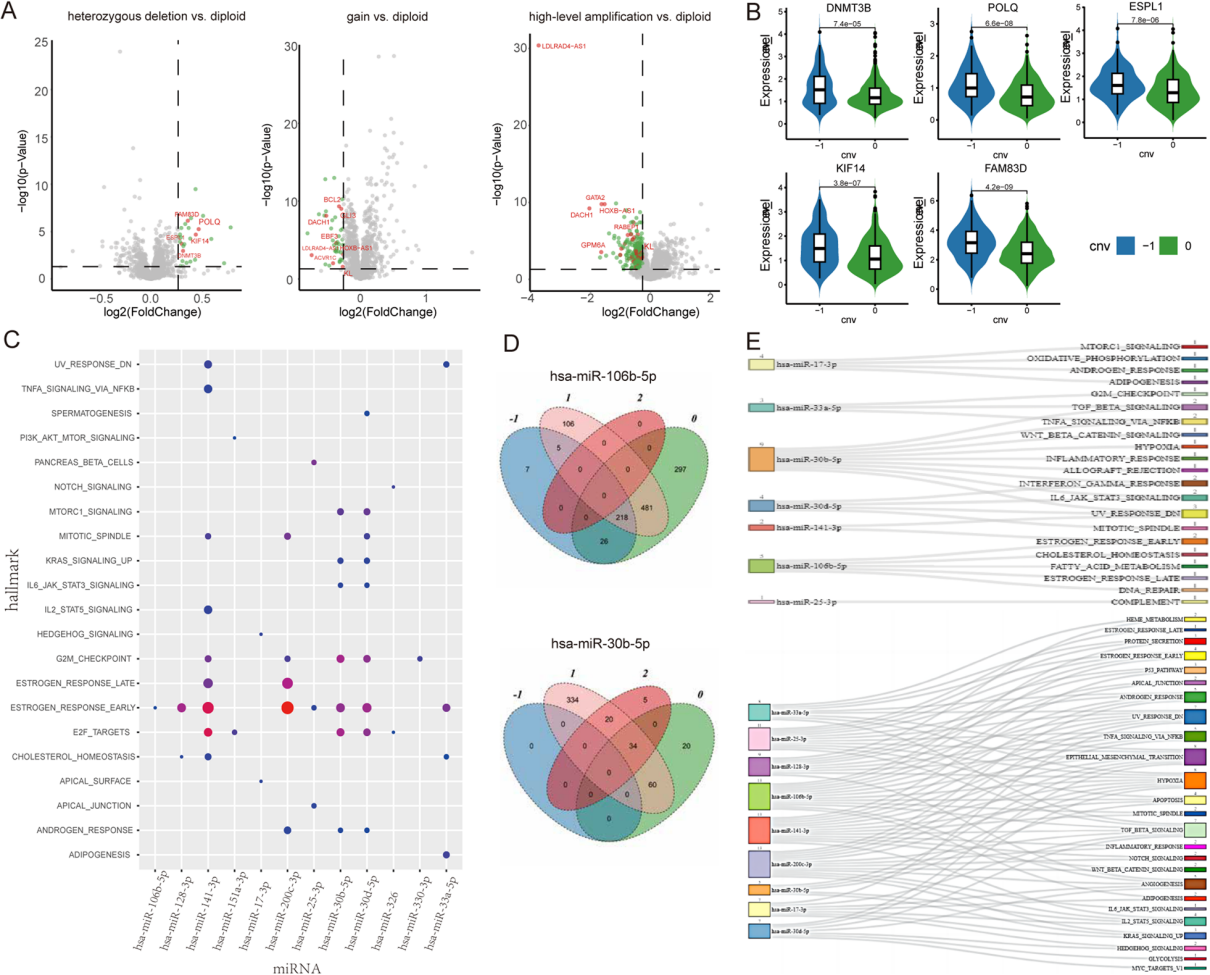
Dynamic target networks of driver miRNAs. **A** Volcano plot for dysregulated expression (|log (FC)|> log (1.2), FDR < 0.05) of targets were caused by SCNAs of hsa-miR-200c-3p; Green points denote genes with significantly dysregulated expression; Red points denote carcinoma related genes; Grey points denote genes with non-significantly dysregulated expression. **B** Expression levels of DNMT3B, POLQ, ESPL1, KIF14 and FAM83D were significantly upregulated by heterozygous deletion of has-miR-200c-3p. **C** The hallmark signatures were significantly enriched by dysregulated expression targets caused by SCNAs of driver miRNAs. **D** Hsa-mir-30b-5p and hsa-mir-106b-5p intersection of active targets under different copy number states. **E** The hallmark signatures were significantly enriched by the dysregulated target networks

For each miRNA, we compared the expression level of target genes and the active miRNA-target relation networks among different copy number states (− 1 vs 0, 1 vs 0 and 2 vs 0). The results showed that 29 candidate miRNAs, whose SCNAs could further cause the dysregulation of target gene expression or imbalance of target networks, were identified as driver miRNAs. The differential expression analysis among different copy number states of miRNA showed the SCNAs of 28 driver miRNAs could cause significantly reverse dysregulation (|log (FC)|> log (1.2), FDR < 0.05) in their targets (Additional file 1: Figure S1). For example, compared with the diploid state, the heterozygous deletion of has-miR-200c-3p significantly up-regulated the expression levels of target genes including DNMT3B, POLQ, ESPL1, KIF14 and FAM83D (Fig. 3A, B). These genes were reported as cancer-related target genes and their overexpression caused poor prognosis in breast cancer patients [19–23]. And the gain and high-level amplification of hsa-miR-200c-3p expression significantly downregulated the expression levels of carcinoma-related genes (Fig. 3A), such as DACH1, which acted as a negative regulator of CD44 to suppress breast cancer [24], and Klotho (KL) was a putative tumor suppressor gene in breast and pancreatic cancers located at chromosome 13q12 [25]. Hallmark functional enrichment analysis showed that the reversely dysregulated expression targets caused by SCNAs of driver miRNAs were significantly enriched in signaling, metabolic, and proliferation hallmark signatures (Fig. 3C). For example, the dysregulated expression targets of hsa-miR-141-3p significantly enriched in ESTROGEN_RESPONSE_EARLY, CHOLESTEROL_HOMEOSTASIS and E2F_TARGETS. Six hallmark signatures (ESTROGEN_RESPONSE_EARLY, E2F_TARGETS, CHOLESTEROL_HOMEOSTASIS, ANDROGEN_RESPONSE, G2M_CHECKPOINT, and MITOTIC_SPINDLE) were repeatedly regulated by at least three driver miRNAs (Details of the enrichment results are shown in Additional file 2: Table S1).

We found that SCNAs of 29 driver miRNAs disturbed the balance of miRNA-target relation networks in two different patterns. Among them, the SCNAs of 25 driver miRNAs severely disrupted the miRNA-mediated active target networks under diploid condition, while the SCNAs of remaining 4 driver miRNAs enhanced the number of active targets in miRNA-mediated target networks (Additional file 1: Figure S2). For example, the active miRNA-target networks mediated by hsa-miR-106b-5p comprised 1,022 active miRNA-target relationships under the diploid state, while only 256 active miRNA-target relations were significantly identified under heterozygous deletion state, and the high-level amplification of has-miR-106b-5p completely destroyed the active miRNA-target relation networks (Fig. 3D). In contrast, the gain of hsa-mir-30b-5p made the number of significantly active miRNA-target relations increase to 448 from 114 in the hsa-mir-30b-5p diploid state (Fig. 3D).

To investigate potential functional disparities among different patterns of dysregulated target networks, we classified these driver miRNA-mediated dysregulated active networks into two categories and performed functional enrichment analysis of cancer hallmarks for two categories. Class I consisted of targets that lose activity due to the SCNAs of driver miRNA, class II consisted of targets that obtained activity due to the SCNAs of driver miRNA. We found that the class I dysregulated active networks consistently and significantly enriched in developmental, pathway and signaling hallmark signatures (Fig. 3E). For example, the most frequently enriched hallmark signatures included EPITHELIAL MESENCHYMAL TRANSITION (8/9), HYPOXIA (8/9), TGF BETA SIGNALING (7/9), TNFA SIGNALING VIA_NFKB (5/9) and APOPTOSIS (4/9), which were enriched by at least four dysregulated active networks. The functional enrichment results of class II dysregulated active target networks showed significant specificity, only UV_RESPONSE_DOWN was enriched by three dysregulated active networks. The class II dysregulated active target networks caused by SCNAs of hsa-miR-30b-5p and hsa-miR-30d-5p were specifically enriched in immune and signaling hallmark signatures, including INTERFERON_GAMMA_RESPONSE, ALLOGRAFT_REJECTION, TNFA_SIGNALING_VIA_NFKB, and WNT_BETA_CATENIN_SIGNALING. However, the class II dysregulated active network caused by SCNAs of hsa-miR-106b-5p was specifically enriched in metabolic and signaling hallmark signatures, including CHOLESTEROL_HOMEOSTASIS, FATTY_ACID_METABOLISM, and ESTROGEN_RESPONSE_LATE (Fig. 3E). (Details of the enrichment results are shown in Additional file 3: Table S2).

Literature search showed that 21 driver miRNAs were recorded in at least one of the known cancer miRNA databases (miRCancer and OncomiRDB) [26, 27] and reported to be associated with breast cancer. Additionally, the remaining 8 driver miRNAs that also have been reported to play important cancer-associated functions in vivo or in vitro (Additional file 4: Table S3). For example, the downregulation of miRNA-141 in breast cancer cells was associated with cell migration and invasion: involvement of ANP32E targeting [28]. The inhibition of MiR-330-3p on target CCBE1 promoted metastasis in human breast cancer [29]. MiR-659-5p was identified as one of the independent risk factors associated with OS time of triple-negative breast cancer [30]. MiR-92a-1-5p could differentiate tumors from normal samples as well as discriminate the molecular subtypes of breast cancer [31]. These findings proved the driver roles of miRNAs with SCNAs.

### Imbalance of ceRNA networks caused by SCNAs of driver miRNAs

**Fig. 4:**
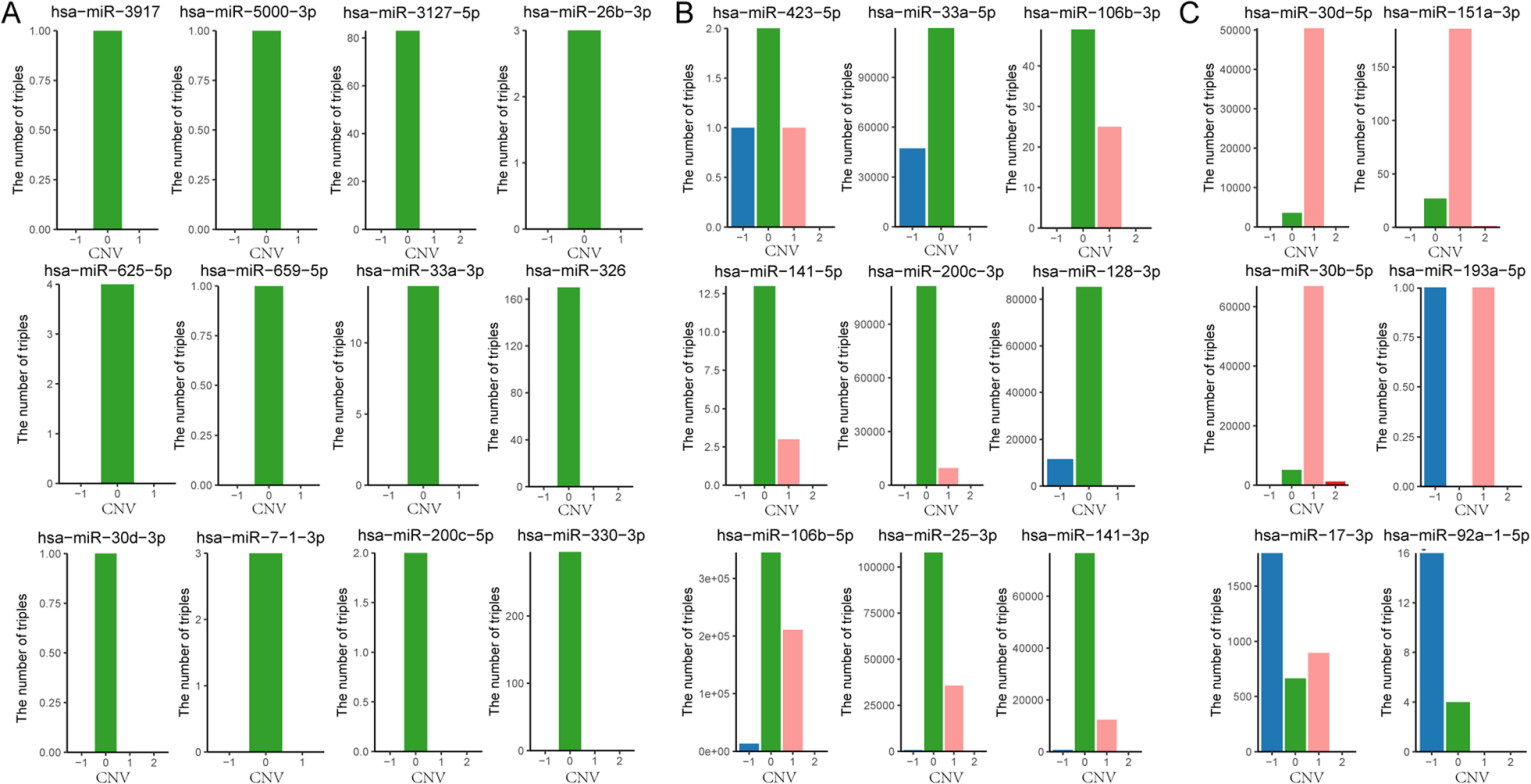
The number of active ceRNA triples in dynamic ceRNA networks under different copy number status for 27 driver miRNAs in BRCA. **A** The active ceRNA triples were destroyed by any copy number alteration of 12 driver miRNAs. **B** The activated ceRNA triples were gradually destroyed with alteration extent of copy number of 9 driver miRNAs. **C** More activated ceRNA triples were obtained with gain or heterozygous deletion of 6 driver miRNAs

Many-to-many target relationships between miRNAs and their targets formed the complex miRNA-mediated ceRNA networks. Abnormal expression of endogenous miRNAs caused by copy number alterations could disrupt the dynamic balance of miRNA-mediated ceRNA networks and drive cancer initiation and progression. To characterize the driver effect of miRNA SCNAs on ceRNA networks, we extracted the static ceRNA networks directly mediated by each driver miRNA from miRNA-target relationships. Based on the expression profiles of PGs, lncRNAs, and miRNAs, we constructed active ceRNA networks mediated by driver miRNAs under each miRNA copy number state using Pearson correlation analysis (FDR = 0.05). We found 27 driver miRNAs mediated different dynamic ceRNA networks under different copy number states (Fig. 4).

We identified three distinct influence patterns of SCNAs of 27 driver miRNAs on their mediated ceRNA networks. The first pattern was that SCNAs of 12 driver miRNAs could severely disrupted the ceRNA networks activated under the diploid condition. For example, the active ceRNA network mediated by has-miR-326, which consisted of 170 ceRNA triples under the diploid state, while none of those ceRNA triples was identified under any SCNA states (Fig. 4A). The second was that the active ceRNA networks mediated by the 9 driver miRNAs are gradually disrupted with the extent of copy number alteration. (Fig. 4B). For instance, the numbers of active ceRNA triples of has-miR-106b-5p decreased from 344,697 to 210,868 and ultimately to 0 as the copy number states changed from diploid to gain and then to high-level amplification. And the third was that only a few active ceRNA triples were identified under the diploid state while these driver miRNAs participated in more active triples under the state of gain or heterozygous deletion (Fig. 4C). Such as has-mir-30b-5p, only 5113 ceRNA triples were significantly activated under the diploid state, while 66,980 active ceRNA triples were identified under the gain state. These results indicated that SCNAs of driver miRNAs could drive the dynamic balance of ceRNA networks by increasing or decreasing their own expression levels, which may contribute to development of cancer.

### High-level amplification of driver miRNAs in 8q24 characterized a BRCA subtype with poor prognosis

**Fig. 5:**
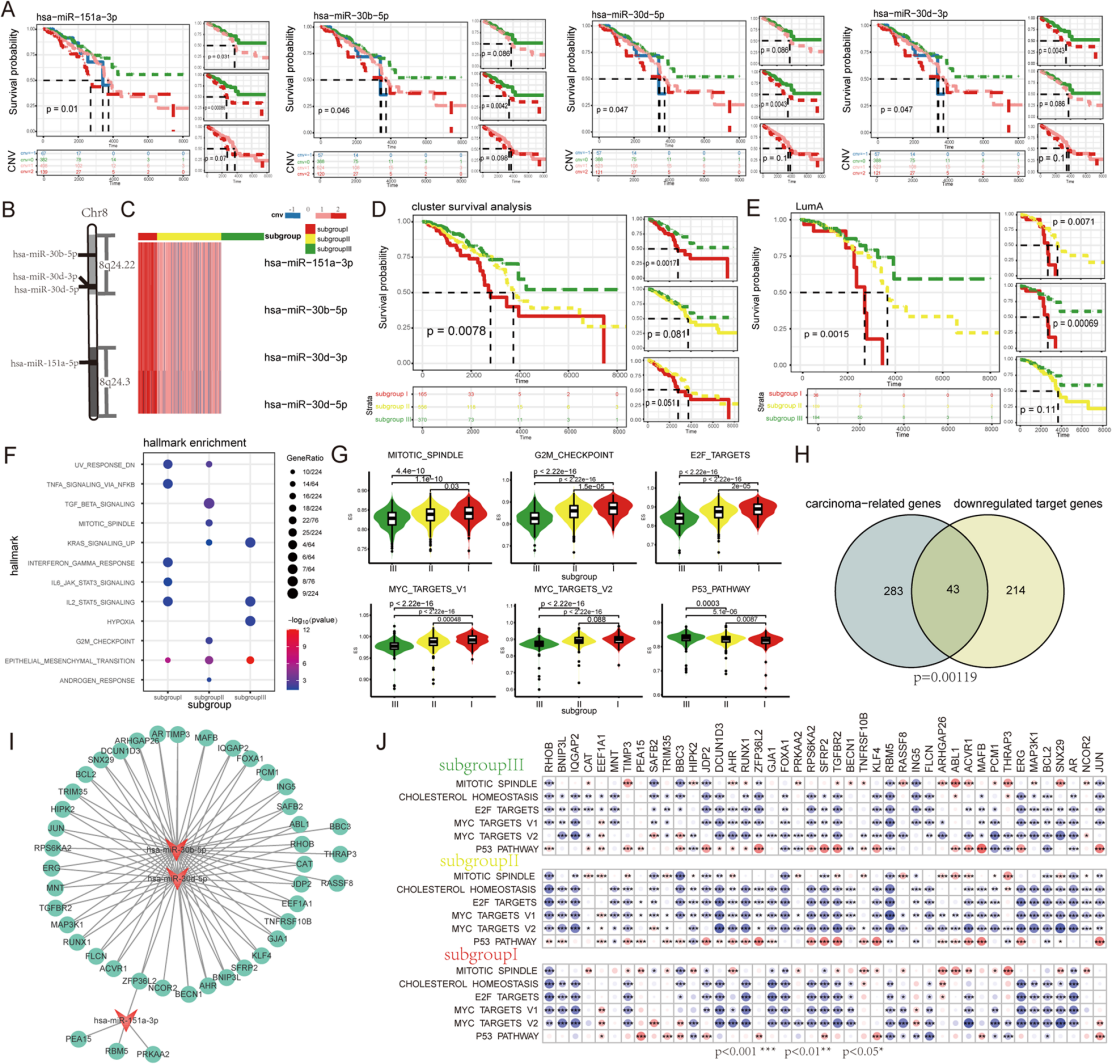
The high-level amplifications of 8p24 characterized a BRCA subtype with poor prognosis. **A** The high-level amplifications of four miRNAs, including has-miR-30d-3p, has-mir-30b-5p, has-miR-30d-5p and has-miR-151a-3p, were associated with poor prognosis in BRCA. **B** Schematic diagram of chromosome location of has-miR-30d-3p, has-mir-30b-5p, has-miR-30d-5p and has-miR-151a-3p in BRCAs. **C** Subgroup clustering SCNAs heatmap of 4 driver miRNAs in BRCA patients. **D** 8q24 amplification characterized a new BRCA subtype with poor prognosis. **E** 8q24 amplification subdivided LumA subtype with significant survival differences. **F** Hallmark signatures significantly were enriched by the dysregulated ceRNA networks of subgroups. **G** The enrichment scores of proliferation markers were significantly higher in subgroup I compared to subgroups II and III. **H** Significantly downregulated target genes in subgroup I significantly overlapped with cancer-related genes. **I** Target network of 43 cancer-related genes regulated by has-mir-30b-5p, has-mir-30d-5p, and has-mir-151a-3p. **J** The correlation heatmap between the expression of cancer-related genes and the activity levels of proliferation hallmarks. Red points denote positive correlation, blue points denote negative correlation; the point size represents the degree of correlation (*p < 0.05; **p < 0.01; ***p < 0.001, independent Student’s t-test)

We next examined whether SCNAs of driver miRNAs were significantly associated with BRCA prognosis. For each driver miRNA, we divided BRCA patient samples into multiple subgroups based on copy number states and used Kaplan–Meier analysis and a log-rank test to perform survival analysis. The results showed that high-level amplification of the four driver miRNAs were significantly associated with poor prognosis of BRCA, including has-miR-30d-3p (*p* = 0.047, log-rank test), has-mir-30b-5p (*p* = 0.046, log-rank test), has-miR-30d-5p (*p* = 0.047, log-rank test) and has-miR-151a-3p (*p* = 0.01, log-rank test) (Fig. 5A).

We investigated the SCNAs distributions of these four driver miRNAs and found that they co-located on 8q24 segment (Fig. 5B), and the high-level amplifications of these four driver miRNAs showed significant co-occurrence patterns (*p* = 1.05e−14, DISCOVER, Fig. 5C). According to the SCNAs of these four driver miRNAs, BRCA patients were subdivided into three subgroups: subgroup I, including patients with high-level amplification in at least one of four driver miRNAs; subgroup II, including patients without high-level amplifications but with heterozygous deletion or gain in four driver miRNAs; and subgroup III, including patients without any SCNAs in four driver miRNAs. Our analysis revealed that the survival time of subgroup I was significantly shorter than those of subgroups II and III (*p* = 0.0078, log-rank test, Fig. 5D). We also observed that the SCNAs of these four driver miRNAs could further subdivide LumA subtype into three subgroups with significantly different survival times, and LumA patients in the subgroup I showed the shortest survival time (Fig. 5E). The high-level amplifications of these four driver miRNAs characterized a new subtype of LumA patients.

To investigate the functional role of the four driver miRNAs in poor prognosis of BRCA, we constructed dynamic ceRNA networks mediated by these miRNAs for each subgroup. We found that the ceRNA dynamic network of subgroup I significantly enriched in immune hallmark signatures (INTERFERON_GAMMA_RESPONSE, *p* = 2.54e−02; IL6_JAK_STAT3_SIGNALING, *p* = 3.73e−02) and DNA damage hallmark signature (UV_RESPONSE_DOWN, *p* = 1.77e−02), which were not enriched in subgroup II or III (Fig. 5F). Additionally, single-sample GSEA (ssGSEA) [32] revealed that the activity levels of proliferation hallmark signatures in subgroup I were significantly higher than those in subgroup II and III (Fig. 5G), such as G2M CHECKPOINT (subgroup I vs III *p* < 2.22e−16, subgroup I vs II *p* = 1.5e−05) and E2F_TARGETS (subgroup I vs III *p* < 2.22e−16, subgroup I vs II *p* = 2e−05).

In order to further elucidate the regulation of these cancer hallmarks by the four driver miRNAs, we used t-test to identify significantly downregulated target genes in subgroup I compared to subgroups II and III (Additional file 1: Figure S3). We found that the downregulated targets genes significantly overlapped with carcinoma-related genes in Tumor suppressor gene (TSGene) [33] and Cancer Gene Census (CGC) [34] databases (*p* = 0.00119, hypergeometric test, Fig. 5H). Specifically, 43 carcinoma-related genes were targeted by has-mir-30b-5p, has-mir-30d-5p, and has-mir-151a-3p (Fig. 5I), and their expression levels were consistently and significantly downregulated in subgroup I. Among these genes, RhoB, BNIP3L and IQGAP2 have been reported as suppressor genes in breast cancer [35–37]; the high expression levels of BCL2 has been reported as an independent indicator of good prognosis in all types of early breast cancer [38]; and the expression silence of Foxa1 has been reported could promote breast cancer invasion and metastasis [39]. Moreover, the expression levels of these carcinoma-related genes exhibited a significant negative correlation with the activity levels of proliferation hallmarks in each subgroup (Fig. 5J), but demonstrated a positive correlation with the activity of the P53_PATHWAY, which is a well-known tumor suppressor [40].

These results suggested that the high-level amplification of these four driver miRNAs inhibited the expression of tumor suppressor genes and further promoted cell proliferation activity to contribute to poor BRCA prognosis.

### Cancer-specific driver SCNA of miRNA

**Fig. 6:**
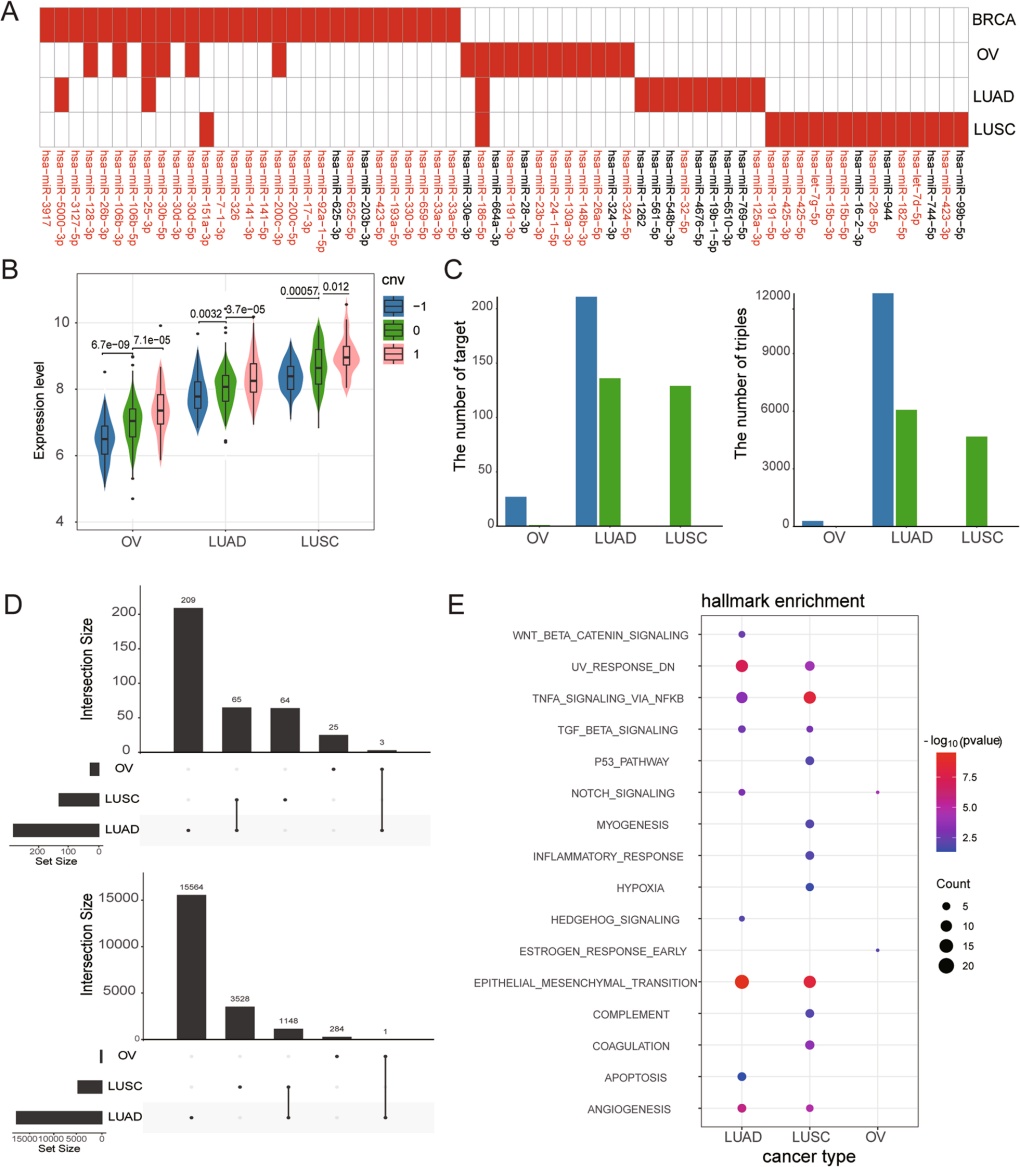
Cancer specificity of the ceRNA networks in multiple cancer types. **A** Heatmap of driver miRNAs across 3 cancer types. **B** Amplification of hsa-mir-186-5p elevated expression levels across three cancers. **C** The number of active targets and active ceRNA triples participated by hsa-mir-186-5p under the conditions of hsa-mir-186-5p diploid and SCNA. **D** Upset plots for dysregulated ceRNA triplets and targets caused by has-186-5p SCNA across the three cancer types. **E** Hallmark signatures were significantly enriched by the dysregulated ceRNA networks caused by SCNAs of hsa-186p-5p across three cancer types

We applied the method to four cancer types [BRCA, Ovarian cancer (OV), Lung adenocarcinoma (LUAD), and Lung Squamous Cell Carcinoma (LUSC)] and identified 64 driver miRNAs. Interestingly, the majority of these driver miRNAs were specific to a particular cancer type, with only a small subset being shared by two or more cancer types (Fig. 6A). Among them, hsa-mir-186-5p was identified as driver miRNA in 3 cancer types (OV, LUAD and LUSC). Numerous studies have reported that overexpression of hsa-miR-186-5p remarkably promoted the proliferation, migration and invasion of LUAD cells [41]. The HOXD-AS1/miR-186-5p/PIK3R3 pathway has been found to play an important role in invasion, migration and EMT of epithelial ovarian cancer [42]. Our analysis revealed that the expression levels of hsa-miR-186-5p significantly increased with the copy number in cancers (*p* value < 0.05) and showed significant differences among the different types of cancers (Fig. 6B).

The SCNAs of hsa-miR-186-5p drove different dysregulated patterns of ceRNA networks across cancer types. In LUSC, the gain and heterozygous deletion of hsa-miR-186-5p completely destroyed the active ceRNA networks under the diploid status. In contrast, in OV, the active ceRNA network contained 285 active ceRNA triples under hsa-miR-186-5p heterozygous deletion state while there were no active ceRNA triples under the diploid state. However, in LUAD, the regulatory effect of hsa-miR-186-5p on active ceRNA networks increased under the heterozygous deletion state compared to the diploid state, and it completely disappeared under gain state (Fig. 6C). By comparing the dysregulated ceRNA networks among different cancer types, we found that the overlaps of dysregulated ceRNA triples and targets was very low (Fig. 6D), with only three targets and one triple being dysregulated in all three cancer types. Surprisingly, the result of functional enrichment analysis shows that dysregulated ceRNA networks of hsa-mir-185-5p in LUAD and LUSC consistently and significantly enriched in signaling, DNA damage and development hallmark signatures, including TNFA_SIGNALING_VIA_NFKB, UV_RESPONSE_DN and EPITHELIAL_MESENCHYMAL_TRANSITION. This suggested that the SCNAs of different driver miRNAs across various cancer types may dysregulate different components of the same cancer hallmark. Moreover, the dysregulated networks in LUSC also specifically enriched in proliferation and immune hallmarks, such as P53_PATHWAY, INFLAMMATORY_RESPONSE, COAGULATION and COMPLEMENT.

**Fig. 7:**
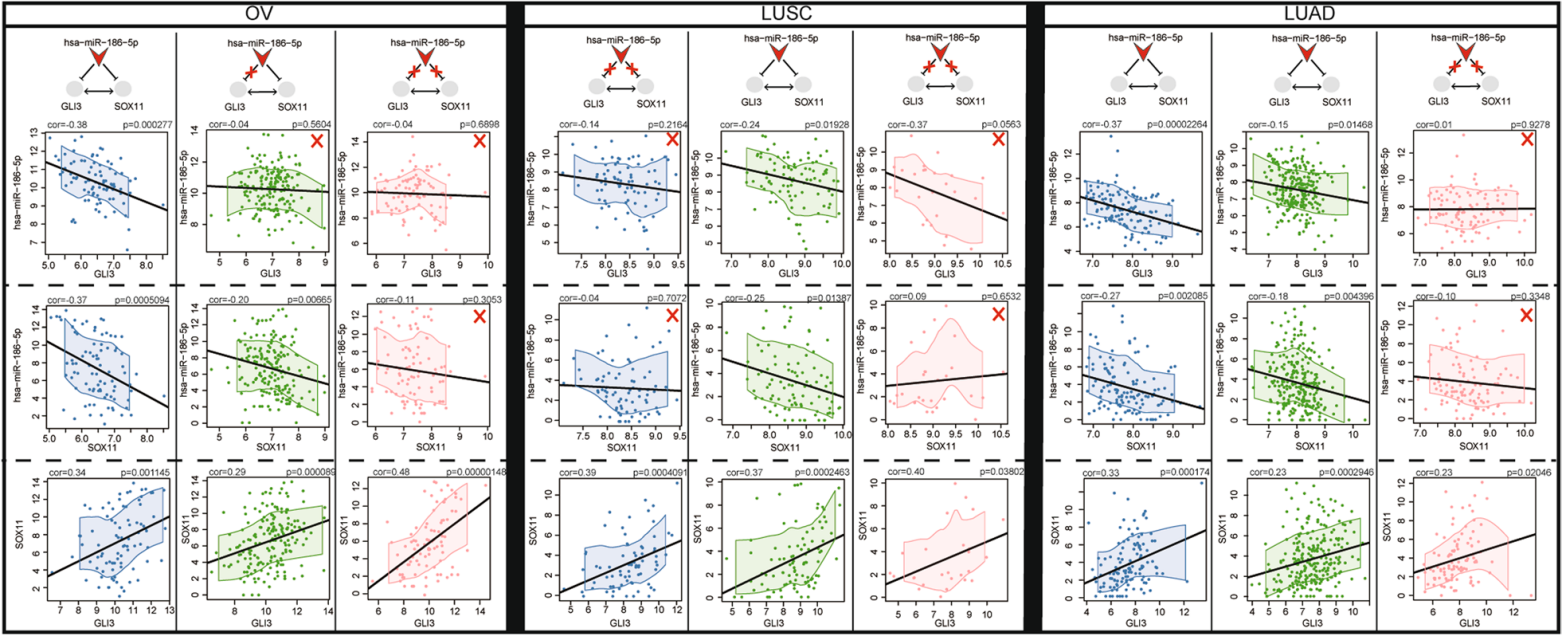
An active ceRNA triple was specific under the different SCNAs of Hsa-miR-186-5p across LUSC, LUAD and OV, which included GLI3 and SOX11, and hsa-miR-186-5p. The blue represents heterozygous deletion, the green represents diploid, and the red represents gain; red cross represents inactive

We also found that differential effects of SCNAs on ceRNA triples across cancer types (Fig. 7). For example, GLI3 and SOX11, which are targeted by hsa-miR-186-5p, formed a stable ceRNA triple. This ceRNA triple was activated under the diploid state of hsa-miR-186-5p in LUSC. GLI3 and SOX11 showed significant positive expression correlation (PCC = 0.37, *p* = 2.46e-04), hsa-miR-186-5p displayed significant negative correlations of expression levels with both GLI3 and SOX11. (PCC = − 0.24, *p* = 0.01928; PCC = -0.25, *p* = 0.01387). However, the significantly active triple relationships were destroyed by any SCNAs of hsa-miR-186-5p in LUSC. In LUAD and OV, this active ceRNA triple was identified under heterozygous deletion of hsa-miR-186-5p. It suggests that the same triple is differentially affected by SCNAs in different cancer types.

These results suggested that the driver SCNA of miRNA contributed to the cancer development by dysregulating different components of cancer hallmarks, suggesting the specificity of driver miRNAs in different cancer types.

## Discussion

MicroRNAs as an important component of non-coding RNAs participate in almost all tumor-related processes by regulating the expression of tumor suppressors and oncogenes. But the biological mechanisms of SCNAs of miRNAs remain poorly understood. In this study, we designed a method to identify driver SCNAs of miRNA and characterized the functional role of miRNA SCNAs through its targets and ceRNA networks across multiple cancer types.

The frequency of miRNA SCNAs is lower than that of genes, with a high-level amplification frequency ranging from 0.5 to 1.6%, and homozygous deletion occurring at an even lower frequency or not detectable in most samples. This requires a sufficient number of samples to support the subsequent analysis of miRNA copy number changes, and it may also be the reason for the function of miRNA SCNAs has been underestimated. In addition, many previous studies of SCNAs have focused solely on homozygous deletions and high-level amplifications as reliable SCNAs, whereas heterozygous deletions and gains were both classified as non-alterations. In our study, we found that heterozygous deletion and gain of miRNAs caused functional dysregulation of the ceRNA networks, which were significantly different from those caused by homozygous deletion and high-level amplification. For example, has-mir-30b-5p participated in more active ceRNA triples under the gain state, while the ceRNA triples were extremely destroyed by high-level amplification. This suggests that heterozygous deletions and gains also played an important role in cancer development and may apply to other non-coding RNAs as well.

MiRNAs are essential negative regulators of mRNAs, capable of regulating gene expression post-transcriptionally by inhibiting mRNA translation or promoting mRNA degradation. In this study, we identified driver SCNAs of miRNAs that drove their own differential expression and further caused the unbalance of target networks or dysregulation of target gene expression. We found that the SCNAs of most drive miRNAs severely destroyed active target networks and caused dysregulated expression of oncogenic and suppressor genes, but a small fraction increased the number of active targets. The dysregulated target networks showed significant enrichment in developmental, pathway, and signaling hallmark signatures for inactivated targets due to SCNAs of driver miRNAs, while activated targets were enriched in immune, metabolic and DNA damage hallmark signatures.

In previous studies, analysis of ceRNA networks mainly focused on changes dominated by lncRNAs/PGs, and often overlooked the role of miRNAs in ceRNA networks. This limited our understanding of the overall complexity of the ceRNA networks. To obtain a more comprehensive view of the functional effects of miRNAs in cancer, we examined the effect of miRNA SCNAs on the stability of ceRNA networks. Our analysis revealed that dysregulated ceRNA networks were significantly influenced by driver SCNAs of miRNA, which contributed an important part to induce dysregulation of ceRNA triplets. However, the construction of ceRNA networks is currently limited by the incompleteness and imprecision of miRNA targeting relationships. With the continuous accumulation of relevant miRNA data and the development of biological techniques, our method can further elucidate the functional roles of miRNAs SCNAs in ceRNA networks.

Notably, we found that the dysregulation of ceRNA network induced by SCNAs of driver miRNAs was associated with poor prognosis of patient. Survival analysis showed that high-level amplification of four driver miRNAs on the 8q.24 segment is significantly associated with poor prognosis in BRCA patients. These SCNAs of the four driver miRNAs could classify LumA subtype into three subgroups with significantly different survival times, where patients in subgroup I showed the shortest survival time. Following functional enrichment analysis, we found that the ceRNA dynamic networks of subgroup I exhibited significant enrichment in immune and DNA damage hallmarks, and the activity levels of proliferation hallmarks in subgroup I were significantly higher than those in subgroup II and III. Moreover, in the dysregulated ceRNA networks of the four driver miRNAs, we observed significant downregulation of several tumor suppressor genes (such as RhoB, BNIP3L, and IQGAP2) in subgroup I. Interestingly, we observed a significant negative correlation between the expression of these cancer-related genes and the activity levels of proliferation hallmarks in each subgroup. These findings have important implications for the diagnosis and treatment of BRCA patients and suggest potential targets for therapeutic intervention.

Our analysis of driver miRNA SCNAs across multiple cancer types revealed that specific miRNA SCNAs events drive different types of cancer. For example, high-level amplification of hsa-miR-106b-5p destroyed the dynamic ceRNA network that was constructed under the diploid state specifically in BRCA. Some miRNAs were also identified as driver SCNAs in multiple cancer types, but their SCNAs performed different functional effects in different cancer types. Such as the gain and heterozygous deletion of hsa-miR-186-5p completely disrupted active ceRNA networks under the diploid state in LUSC, while the regulatory effect of hsa-miR-186-5p on active ceRNA networks increased under the state of heterozygous deletion in LUAD. The diversity of miRNA SCNAs across multiple cancers allowed us to explore the cancer specific functions of miRNAs in a more comprehensive view.

## Conclusion

In summary, we provided a method to elaborate the functional mechanism of SCNAs of miRNAs in cancer based on the dysregulated ceRNA networks. Our approach describes the biological function of miRNAs and their SCNAs in a novel perspective. Through survival analysis, we found high-level amplification of the four driver miRNAs (including has-miR-30d-3p, has-mir-30b-5p, has-miR-30d-5p and has-miR-151a-3p) in 8q24 characterized a BRCA subtype with poor prognosis and contributed to the dysfunction of cancer-associated hallmarks in a complementary way. Then, we applied the method to other three cancer types (OV, LUAD and LUSC) and compared the difference of dysregulated ceRNA network driven by SCNAs of driver miRNAs. The result revealed that specific miRNA SCNA events drive different types of cancer and SCNAs of the same miRNA to performed different functional effects in different cancer types.

### Supplementary Information

**Additional file 1. Figure S1.** Volcano plot for dysregulated expression (|log (FC)|>log (1.2), FDR<0.05) of 28 driver miRNAs targets were caused by SCNAs.Green points denote genes with significantly dysregulated expression; red points denote carcinoma related genes; grey points denote genes with non-significantly dysregulated expression. **Figure S2**. The number of active targets in dynamic ceRNA networks under different copy number status for 29 driver miRNAs in BRCA. **Figure S3**. Significantly downregulated target genes in subgroup I compared to subgroups II and III from dynamic ceRNA networks mediated by these miRNAs for each subgroup.(A) Volcano plot for differential expression (|log (FC)|<0, FDR<0.05) of targets between subgroup I and subgroup II. (B) Volcano plot for differential expression (|log (FC)|<0, FDR<0.05) of targets between subgroup I and subgroup III. Blue points denote genes with significantly downregulated expression; red points denote carcinoma related genes; grey points denote genes with non-significantly downregulated expression.

**Additional file 2. Table S1. **Hallmark functional enrichment of reversely dysregulated expression targets induced by SCNA driving miRNA.

**Additional file 3. Table S2. **Hallmark functional enrichment analysis of dysregulated active targets of driver miRNAs.

**Additional file 4. Table S3. **Reported driver miRNAs that play important cancer-related functions in vivo or in vitro.

## Data Availability

For TCGA cohorts, the genomic and clinical data can be retrieved from NCI Genomic Data Commons [NCI-GDC: https://gdc.cancer.gov]. Data of the BRCA, LAUD, LUSC and OV cohort can be obtained from the cBioPortal [https://www.cbioportal.org/]. The miRNA-mRNA target interactions from starBase v2.0 [https://starbase.sysu.edu.cn/index.php] and the miRNA–lncRNA target interactions from lnCeDB [http://gyanxet-beta.com/lncedb/].
