# Supplementary material for: Identifying the driver miRNAs with somatic copy number alterations driving dysregulated ceRNA networks in cancers
Source: Biol Direct. 2023 Nov 22;18:79. doi: 10.1186/s13062-023-00438-x (PMC10666415; doi:10.1186/s13062-023-00438-x)
Supplement: Supplementary file 1 — Additional file 1. Figure S1. Volcano plot for dysregulated expression (|log (FC)|>log (1.2), FDR<0.05) of 28 driver miRNAs targets were caused by SCNAs.Green points denote genes with significantly dysregulated expression; red points denote carcinoma related genes; grey points denote genes with non-significantly dysregulated expression. Figure S2. The number of active targets in dynamic ceRNA networks under different copy number status for 29 driver miRNAs in BRCA. Figure S3. Significantly downregulated target genes in subgroup I compared to subgroups II and III from dynamic ceRNA networks mediated by these miRNAs for each subgroup.(A) Volcano plot for differential expression (|log (FC)|<0, FDR<0.05) of targets between subgroup I and subgroup II. (B) Volcano plot for differential expression (|log (FC)|<0, FDR<0.05) of targets between subgroup I and subgroup III. Blue points denote genes with significantly downregulated expression; red points denote carcinoma related genes; grey points denote genes with non-significantly downregulated expression. [file 13062_2023_438_MOESM1_ESM.docx]

Supplementary Figures


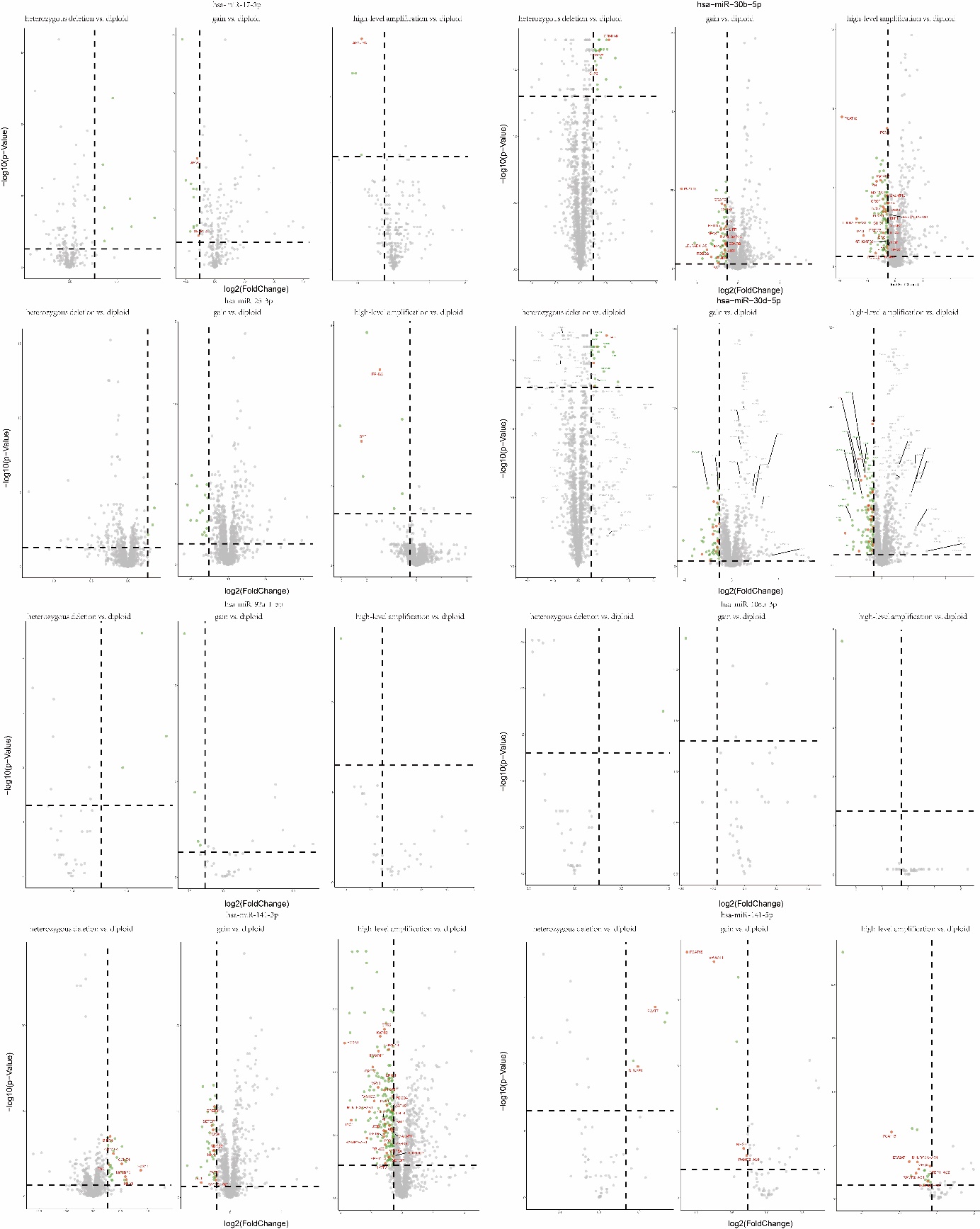


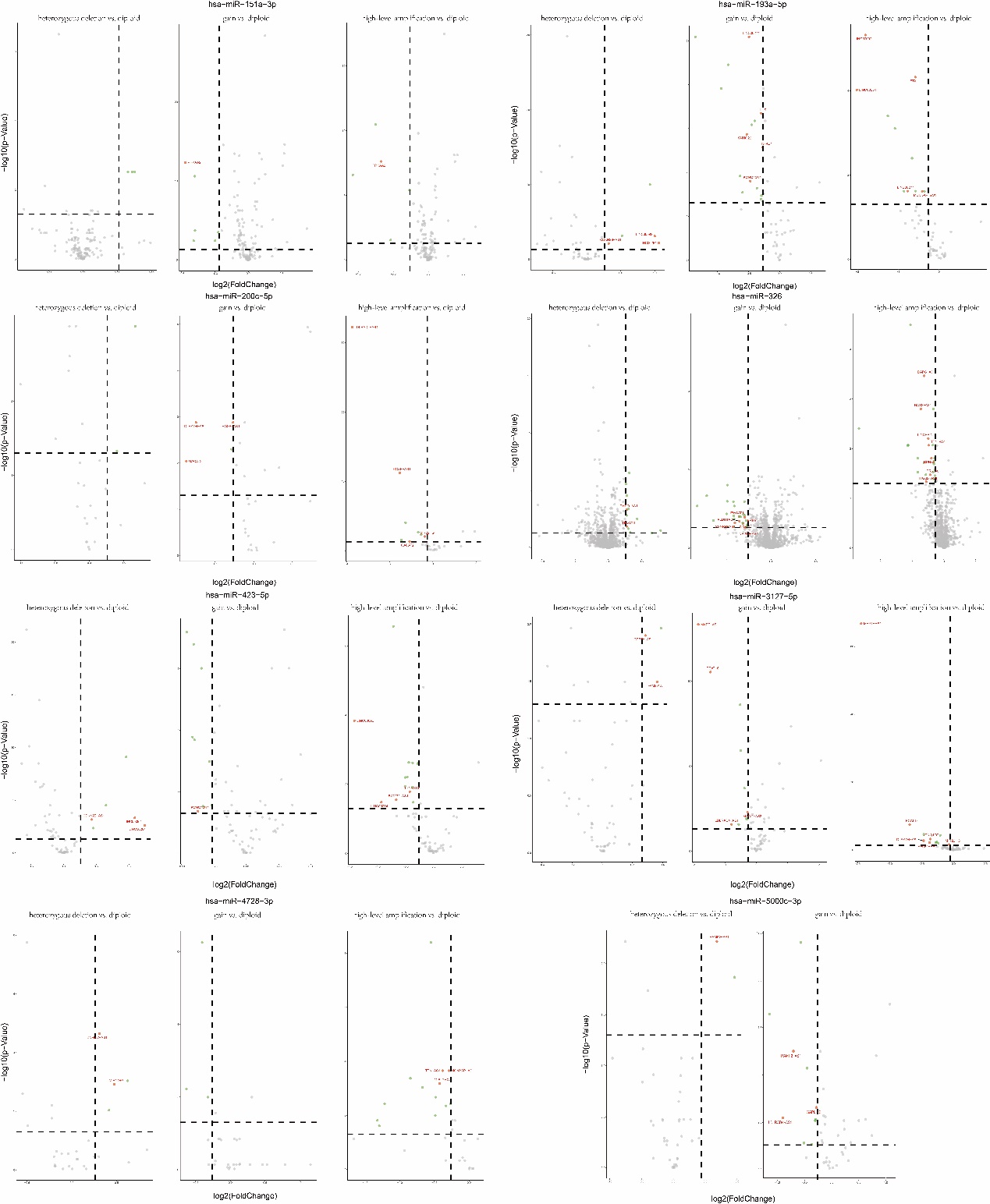


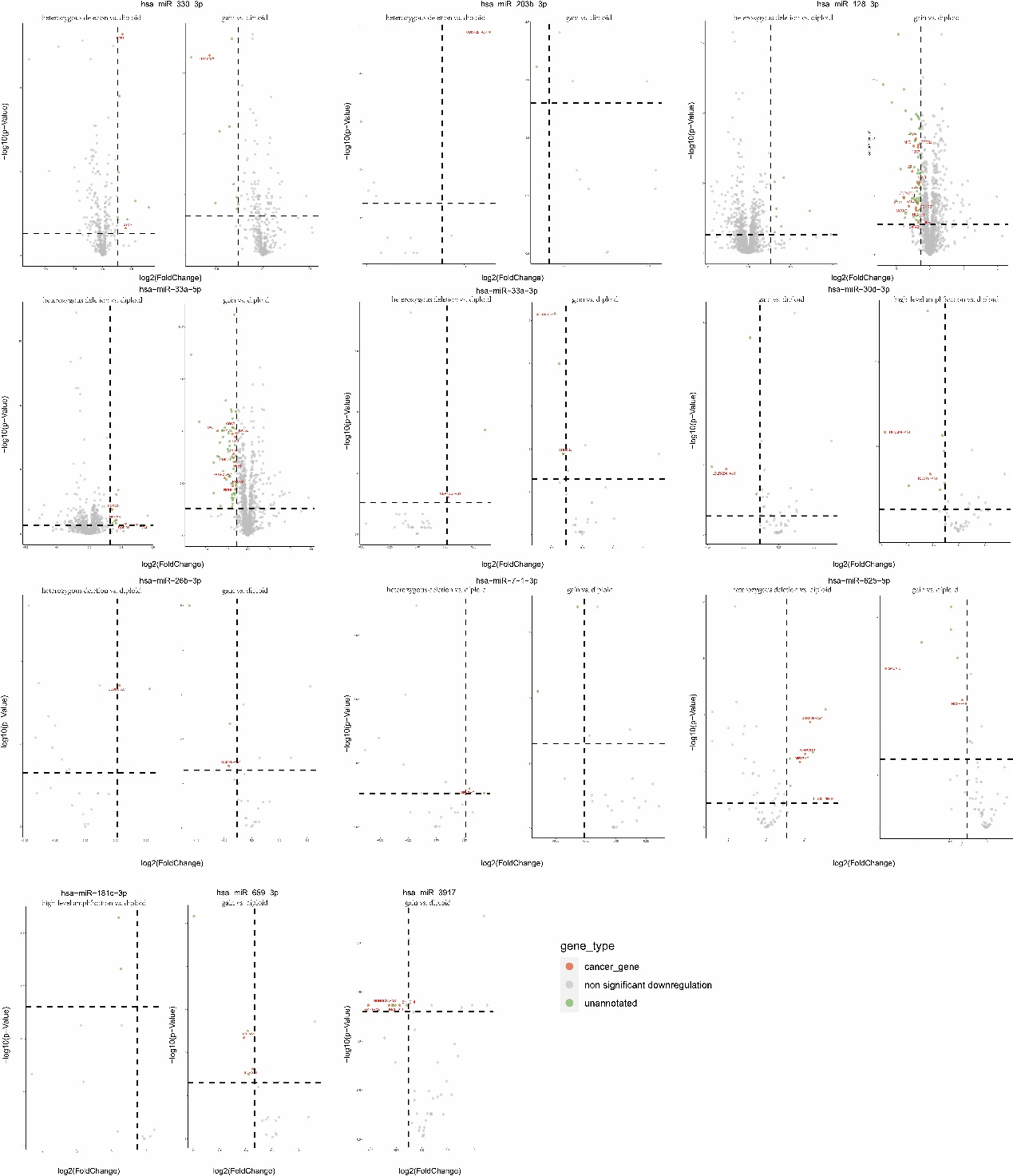


**Supplementary Fig. 1.** Volcano plot for Dysregulated expression (|log (FC)|>log (1.2), FDR<0.05) of 28 Driver miRNAs Targets were Caused by SCNAs.

Green points denote genes with significantly dysregulated expression; Red points denote carcinoma related genes; Grey points denote genes with non-significantly dysregulated expression.

**
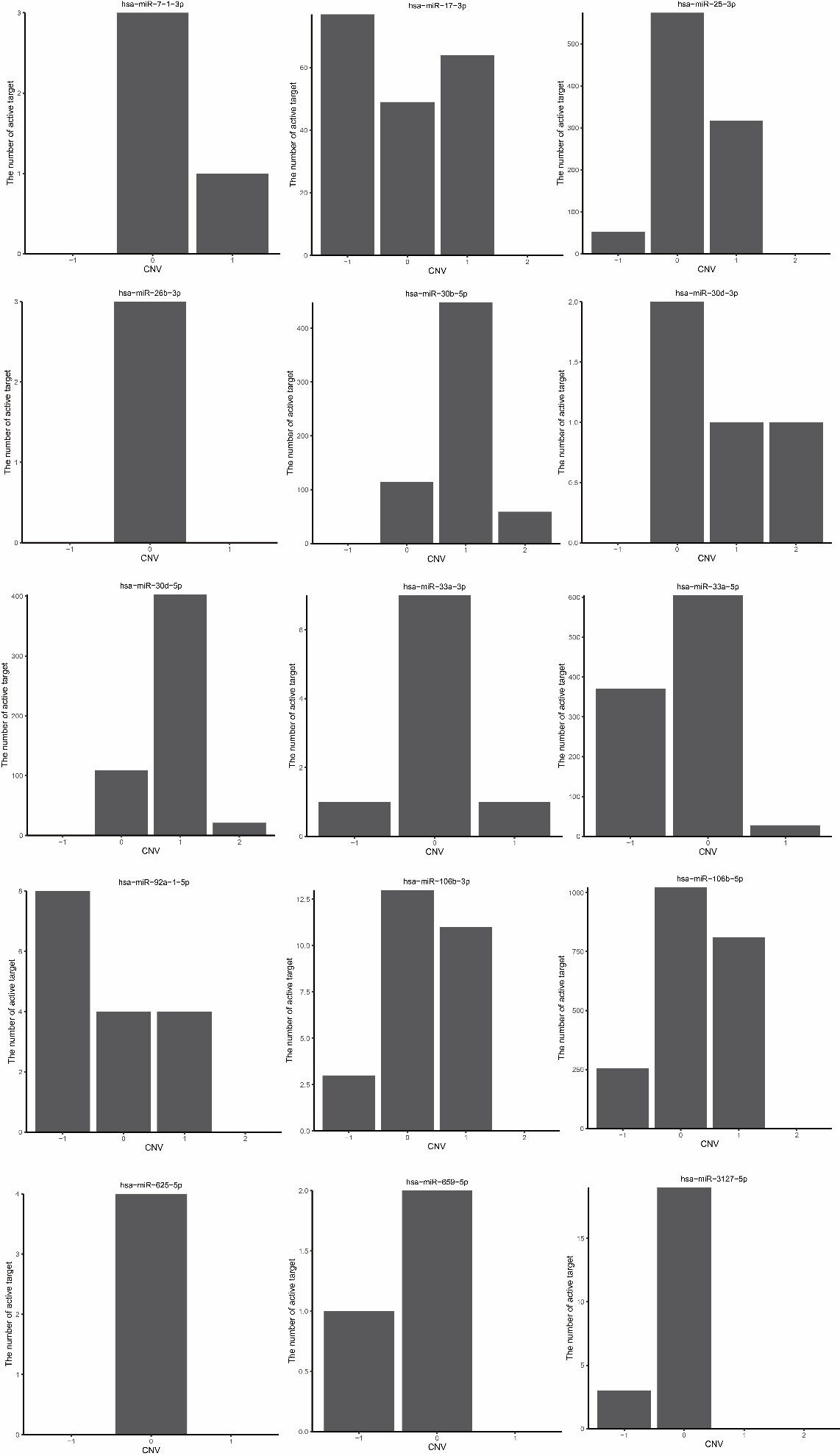
**

**
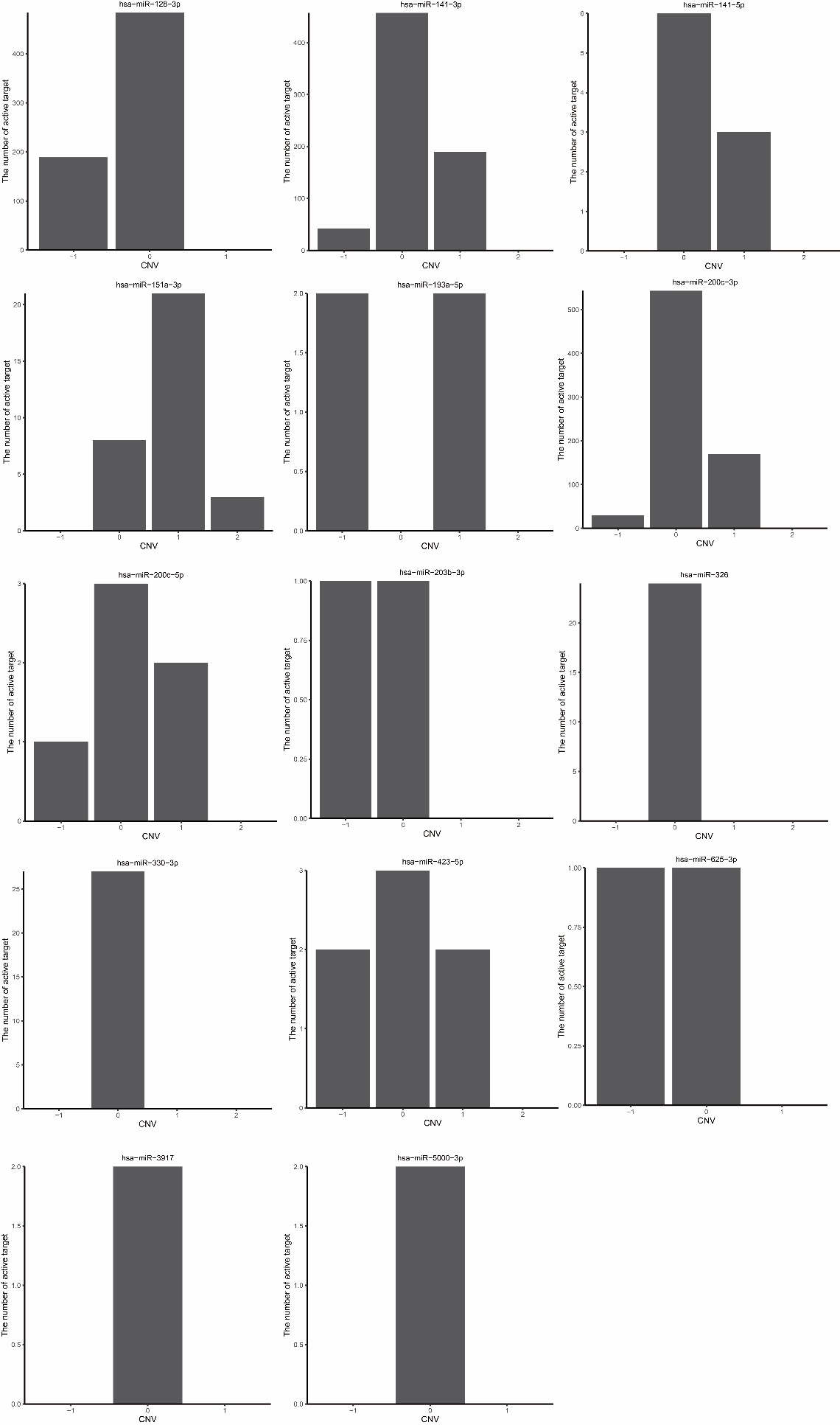
**

**Supplementary Fig. 2.** The Number of Active Targets in Dynamic ceRNA Networks under Different Copy Number Status for 29 Driver miRNAs in BRCA.

**
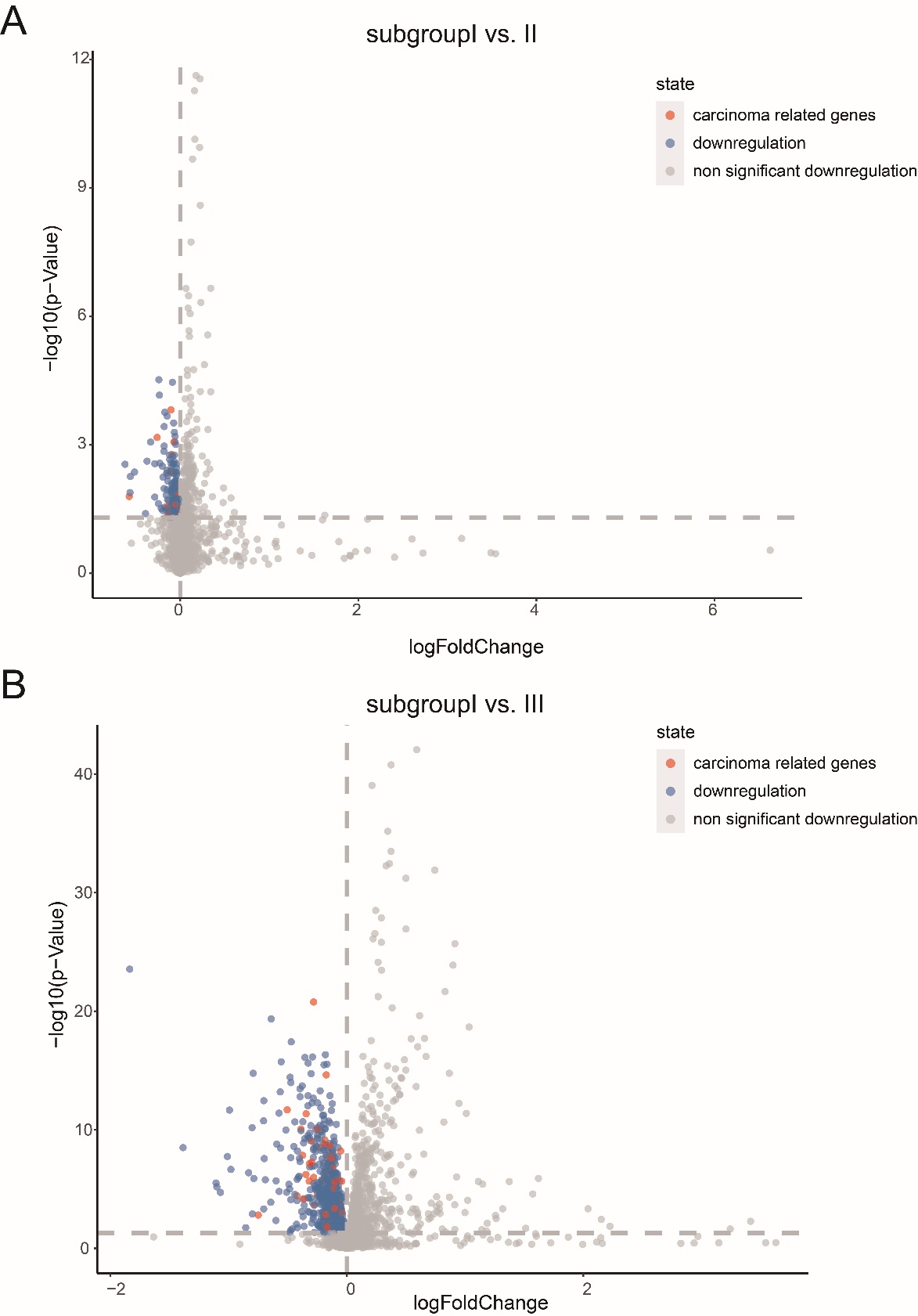
**

**Supplementary Fig. 3.** Significantly Downregulated Target Genes in subgroup I Compared to subgroups II and III from Dynamic ceRNA Networks Mediated by these miRNAs for Each Subgroup.

(A) Volcano plot for differential expression (|log (FC)|<0, FDR<0.05) of targets between subgroup I and subgroup II. (B) Volcano plot for differential expression (|log (FC)|<0, FDR<0.05) of targets between subgroup I and subgroup III. Blue points denote genes with significantly downregulated expression; Red points denote carcinoma related genes; Grey points denote genes with non-significantly downregulated expression.
